# Detection of Acute HIV Infection in Two Evaluations of a New HIV Diagnostic Testing Algorithm — United States, 2011–2013

**Published:** 2013-06-21

**Authors:** Kara Geren, Eric Moore, Cheri Tomlinson, Dan Hobohm, Ann Gardner, Deborah Reardon-Maynard, Cindy Gay, Lisa B. Hightow-Weidman, Mark W. Pandori, Nicholas Moss, Emily Westheimer, Benjamin Tsoi, Bernard M. Branson, Philip J. Peters

**Affiliations:** Maricopa Integrated Health Systems, Phoenix; Arizona Dept of Health Svcs; Univ of North Carolina at Chapel Hill; San Francisco Dept of Public Health; New York City Dept of Health and Mental Hygiene, New York; Div of HIV/AIDS Prevention, National Center for HIV/AIDS, Viral Hepatitis, STD, and TB Prevention, CDC

The highly infectious phase of acute human immunodeficiency virus (HIV) infection, defined as the interval between the appearance of HIV RNA in plasma and the detection of HIV-1–specific antibodies, contributes disproportionately to HIV transmission (1). The current HIV diagnostic algorithm consists of a repeatedly reactive immunoassay (IA), followed by a supplemental test, such as the Western blot (WB) or indirect immunofluorescence assay (IFA). Because current laboratory IAs detect HIV infection earlier than supplemental tests, reactive IA results and negative supplemental test results very early in the course of HIV infection have been erroneously interpreted as negative (2). To address this problem, CDC has been evaluating a new HIV diagnostic algorithm (3). This report describes two evaluations of this algorithm. An HIV screening program at a Phoenix, Arizona emergency department (ED) identified 37 undiagnosed HIV infections during July 2011–February 2013. Of these, 12 (32.4%) were acute HIV infections. An ongoing HIV testing study in three sites identified 99 cases with reactive IA and negative supplemental test results; 55 (55.6%) had acute HIV infection. CDC and many health departments recognize that confirmatory supplemental tests can give false-negative results early in the course of HIV infection. This problem can be resolved by testing for HIV RNA after a reactive IA result and negative supplemental test result.

Early HIV IAs used either viral lysate antigens (first generation) or synthetic peptides and recombinant antigens (second generation) and detected only immunoglobulin G (IgG)-class antibodies. Most laboratories now use either third-generation IAs that detect both immunoglobulin M-class and IgG-class antibodies or fourth-generation combination antigen/antibody IAs that detect both classes of antibody and also p24 antigen (a major core protein of HIV). The p24 antigen can be detected early, before antibody appears, allowing the fourth-generation IAs to identify some HIV infections in the acute phase. In this report, fourth-generation, IA-reactive specimens with a negative supplemental test but detectable HIV-1 RNA were classified as acute HIV infection.

The current laboratory diagnostic algorithm for HIV cannot detect acute infections and misclassifies approximately 60% of HIV-2 infections as HIV-1, based on HIV-1 WB results (4). The new diagnostic algorithm evaluated in this study replaces the WB with an HIV-1/HIV-2 antibody differentiation assay as the supplemental test and includes an RNA test to resolve reactive IA with negative supplemental test results (Figure 1). In retrospective studies, this algorithm performed better than the WB at identifying HIV-antibody–positive persons, detecting acute HIV-1 infections, and diagnosing unsuspected HIV-2 infections (5,6). In this report, data from two evaluations of this algorithm are analyzed, one from an HIV testing program in Phoenix, Arizona, and the other from an ongoing HIV testing study in three sites.

In 2011, the Arizona Department of Health Services collaborated with Maricopa Integrated Health Systems* to 1) screen all adult ED patients (aged 18–64 years) for HIV who had phlebotomy for other reasons as a part of their medical care and 2) validate the new algorithm. Specimens were screened with a fourth-generation IA (Architect HIV Ag/Ab Combo Assay [Architect], Abbott Diagnostics) from July 2011 through February 2013. From July 2011 through February 2012, 10 specimens with repeatedly reactive Architect results were tested with both a WB and a Food and Drug Administration (FDA)-approved HIV-1/HIV-2 antibody differentiation assay (Multispot HIV-1/HIV-2 Rapid Test [Multispot], Bio-Rad Laboratories), and from March 2012 through February 2013, only with a Multispot (27 specimens). Specimens negative by either WB or Multispot were tested for HIV-1 RNA (m2000 RealTime HIV-1 Quantitative Assay, Abbott Diagnostics).

The Screening Targeted Populations to Interrupt On-going Chains of HIV Transmission with Enhanced Partner Notification (STOP) study is evaluating 1) methods to detect acute HIV infection and enhance partner services in New York, New York; North Carolina; and San Francisco, California, and 2) the new diagnostic algorithm. Participants aged >12 years who received HIV testing at one of 12 venues from September 2011 through September 2012 were screened with Architect. Repeatedly reactive specimens were tested with Multispot and either an HIV-1 WB (Bio-Rad Laboratories) or an in-house IFA. Specimens with negative Multispot, WB, or IFA results were tested for HIV-1 RNA (either Aptima HIV-1 RNA Qualitative Assay [Gen-Probe] or m2000 RealTime HIV-1 Quantitative Assay).

Routine HIV screening with Architect in the Phoenix ED from July 2011 through February 2013 detected previously undiagnosed HIV infection in 37 patients (Table). The diagnosis of acute HIV infection was established by a negative supplemental test but a detectable HIV-1 RNA in 12 (32.4%) of these 37 patients. The other 25 HIV diagnoses were antibody-positive by Multispot, WB, or both. The median HIV-1 viral load among patients with acute infection was 3,636,176 copies/mL (interquartile range: 614,164 to >10,000,000), compared with 27,125 copies/mL (9,519–78,084) among patients with established infection.

In the STOP study, Architect results were repeatedly reactive in 654 (1.7%) of 37,876 patients screened from September 2011 through September 2012 (Figure 2). Multispot was reactive for HIV-1 in 554 (84.7%) patients and for both HIV-1 and HIV-2 in one (0.2%). In the 99 (15.1%) patients with a negative or HIV-1 indeterminate Multispot result, HIV-1 RNA was present in 55 (55.6%), representing 8.4% of all those with repeatedly reactive Architect results. Traditional supplemental tests (either HIV-1 WB or IFA) were negative in 37 (67.3%) and indeterminate in seven (12.7%) of these 55 Architect-reactive specimens from patients with acute HIV-1 infection (Figure 2).

## Editorial Note

Improved HIV IAs enhance the ability to detect HIV infection earlier, even during the acute phase of infection, when substantial HIV transmission occurs. However, specimens with reactive IA and negative supplemental test results must undergo further testing to differentiate acute HIV infection from false-positive results. This report demonstrates that acute HIV infections detected with third- or fourth-generation IAs often are misclassified as HIV-negative by WB or IFA, potentially leading to adverse clinical outcomes for patients and further HIV transmission within the community (1). Applying the HIV testing algorithm evaluated in this analysis averted missed diagnoses in 32% of the HIV-infected patients in the Phoenix ED and 9% of those in the STOP study. With FDA’s approval of the Multispot HIV-1/HIV-2 rapid test for use as the second test in this algorithm in March 2013, laboratories can adopt this algorithm, which is a recommended option in the Clinical and Laboratory Standards Institute’s *Criteria for Laboratory Testing and Diagnosis of Human Immunodeficiency Virus Infection; Approved Guideline* (7). The fast turnaround time for test results from most third- and fourth-generation IAs (<1 hour) and the Multispot rapid test (15 minutes) affords the opportunity to deliver same-day definitive test results to the majority of HIV-infected persons who are antibody-positive. Regardless of which supplemental test is used, clinicians and laboratories might want to consider further HIV RNA testing for patients whose supplemental antibody test results are negative after a reactive third- or fourth-generation IA result (8).

The ED at Maricopa Integrated Health Systems adopted routine, opt-out HIV screening consistent with CDC’s 2006 recommendations (9), using a fourth-generation IA. As a result, an additional 37 patients with HIV infection, including 12 with acute infection, were identified. Because most currently available FDA-approved rapid HIV tests are second-generation format (i.e., they detect only IgG-class antibodies), these acute HIV infections likely would have been missed if point-of-care rapid tests had been used for screening. The high percentage of HIV infections that were acute among these ED patients was unexpected; however, consistent with observations that 50%–90% of persons with acute HIV infection develop symptoms that prompt them to seek medical care (10), this finding suggests that acute HIV infection in persons who seek care for its nonspecific symptoms in EDs and other urgent-care venues might go undiagnosed unless HIV screening is conducted with fourth-generation HIV IAs. Currently, only one RNA assay, the Aptima HIV-1 RNA Qualitative Assay, is FDA-approved for HIV diagnosis, but it is available in far fewer laboratories than quantitative HIV-1 (viral load) RNA assays. To facilitate prompt diagnosis of acute HIV infection when faced with discordant screening and supplemental antibody test results, clinicians can order a viral load test to differentiate acute HIV-1 infection from false-positive IA results.

What is already known on this topic?

The highly infectious phase of acute human immunodeficiency virus (HIV) infection, before the appearance of HIV-1-specific antibodies, contributes disproportionately to HIV transmission. Improved HIV laboratory immunoassays (IAs) can detect HIV infection during this acute phase, when traditional HIV supplemental tests (e.g., Western blot) are still negative. Some discordant HIV test results (reactive IA and negative supplemental test) have been erroneously interpreted as HIV-negative.

What is added by this report?

Using an HIV testing algorithm that included RNA testing for all specimens with reactive IA and negative supplemental antibody test results led to the diagnosis of acute HIV infections in various HIV testing settings. Using an HIV IA to screen patients in an Arizona emergency department identified 37 undiagnosed HIV infections, of which 32.4% were acute and would have been misclassified as HIV-negative by current testing practices that rely on antibody tests such as Western blot. An ongoing multisite study of a convenience sample of persons at high risk identified 99 cases with reactive IA and negative supplemental test results; 44.4% were in patients who were not infected, but 55.6% had acute HIV infection. These acute HIV infections would have been misclassified as HIV-negative without RNA testing, potentially leading to adverse clinical outcomes for patients and further HIV transmission within the community.

What are the implications for public health practice?

For patients with a reactive HIV IA result and negative supplemental antibody test results, additional testing for HIV-1 RNA is necessary to identify patients with acute HIV infection. If RNA testing is not available, a follow-up IA should be conducted in 2–4 weeks.

The findings in this report are subject to at least two limitations. First, results might not be generalizable to all HIV screening programs. Although the goal of the Phoenix ED was to screen for HIV as many patients as possible, HIV tests might have been ordered on some patients because of clinical suspicion, potentially increasing the number of HIV or acute HIV infections identified. Second, participants in the STOP study were a convenience sample of persons at high risk for HIV infection attending sexually transmitted infection clinics or community-based HIV testing programs serving men who have sex with men. Therefore, the percentage of HIV-1 infections that were acute might be higher than that observed in other populations.

Third- and fourth-generation IAs are important advances for HIV testing that improve the ability to detect HIV infections earlier. In the two prospective evaluations described in this report, the new diagnostic testing algorithm performed better than the current algorithm for identifying HIV infections. CDC’s recommendation for a new HIV diagnostic algorithm, which will incorporate the findings of this analysis, is under development. Clinicians can use the findings from this report by remaining vigilant for discordant IA and supplemental test results and either ordering an HIV-1 nucleic acid test or obtaining follow-up HIV testing (in 2–4 weeks) to accurately determine whether HIV infection is present.

## Figures and Tables

**FIGURE 1:**
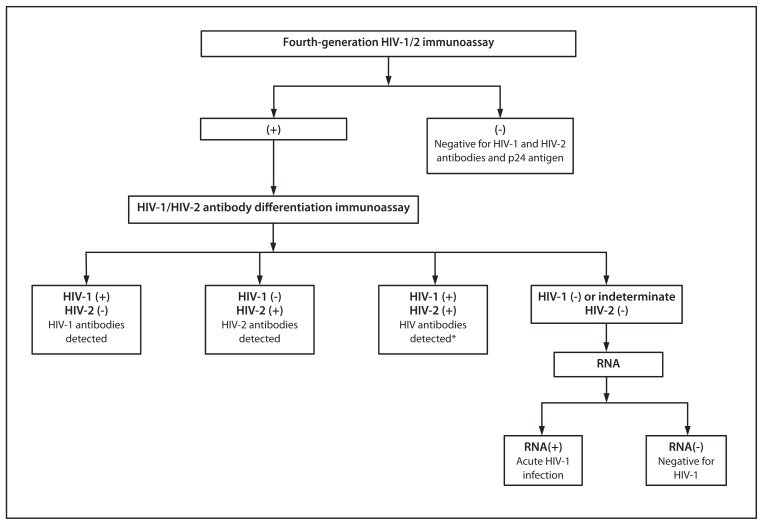
New HIV diagnostic testing algorithm evaluated — United States, 2011–2013 **Abbreviation:** HIV = human immunodeficiency virus. * Additional testing required to rule out dual infection with HIV-1 and HIV-2.

**FIGURE 2:**
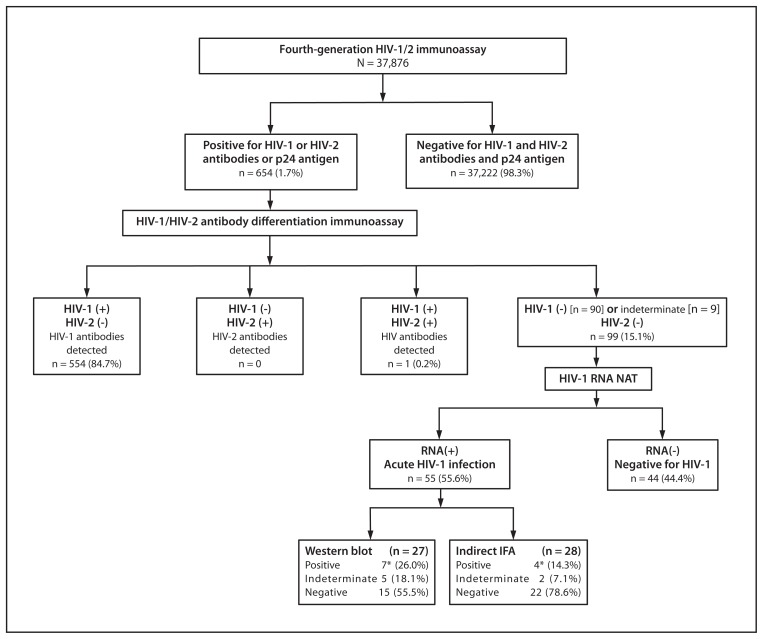
Fourth-generation HIV-1/2 immunoassay test results with the new HIV diagnostic testing algorithm — New York, New York; San Francisco, California; and North Carolina, September 2011–2012 **Abbreviations:** HIV = human immunodeficiency virus; NAT = nucleic acid test; IFA = immunofluorescence assay. * Five of the seven Western blot positive results and two of the four IFA positive results occurred with specimens that were HIV-1 indeterminate on the differentiation assay. The differentiation assay has four reaction spots, including 1) control, 2) HIV-2 peptide, 3) recombinant HIV-1, and 4) HIV-1 peptide. When used in a diagnostic algorithm, both HIV-1 spots (recombinant and peptide) must be reactive for a specimen to be interpreted as positive for HIV-1 antibodies. The presence of only one HIV-1 spot is interpreted as indeterminate for HIV-1 antibodies.

**TABLE:** Demographic characteristics, clinical symptoms, and HIV test results of patients who had HIV infection diagnosed in an emergency department (ED) using a reactive fourth-generation immunoassay — Phoenix, Arizona, 2011–2013

Patient	Sex	HIV infection status	ED encounter date	Differentiation IA	Western blot	HIV-1 viral load (RNA copies/mL)
Patient 7	Male	Acute	Oct 2011	Nonreactive	Negative	>10,000,000
Patient 8	Male	Acute	Dec 2011	Nonreactive	Negative	5,370,318
Patient 11	Male	Acute	Jan 2012	Nonreactive	Inconclusive	1,141,782
Patient 19	Female	Acute	Apr 2012	Nonreactive	ND	>10,000,000
Patient 25	Male	Acute	Jun 2012	Nonreactive	ND	>10,000,000
Patient 36	Male	Acute	Sep 2012	Nonreactive	ND	>10,000,000
Patient 23	Male	Acute	May 2012	Nonreactive	ND	4,357,922
Patient 39	Male	Acute	Sep 2012	Nonreactive	ND	691,343
Patient 57	Male	Acute	Jan 2013	Nonreactive	ND	382,628
Patient 31	Female	Acute	Jul 2012	Nonreactive	ND	309,139
Patient 27	Male	Acute	Jun 2012	Nonreactive	ND	64,163
Patient 3	Male	Acute	Aug 2011	HIV-1 reactive	Negative	2,914,430
Patient 13	Male	Established	Jan 2012	HIV-1 reactive	Positive	86,910
Patient 6	Male	Established	Oct 2011	HIV-1 reactive	Positive	29,476
Patient 5	Female	Established	Oct 2011	HIV-1 reactive	Positive	18,822
Patient 4	Male	Established	Sep 2011	HIV-1 reactive	Positive	15,608
Patient 12	Male	Established	Jan 2012	HIV-1 reactive	Positive	11,209
Patient 2	Male	Established	Aug 2011	HIV-1 reactive	Positive	6,460
Patient 40	Female	Established	Sep 2012	HIV-1 reactive	ND	<40
Patient 56	Male	Established	Jan 2013	HIV-1 reactive	ND	764,498
Patient 32	Male	Established	Aug 2012	HIV-1 reactive	ND	690,951
Patient 16	Male	Established	Mar 2012	HIV-1 reactive	ND	632,488
Patient 59	Male	Established	Feb 2013	HIV-1 reactive	ND	602,878
Patient 42	Male	Established	Oct 2012	HIV-1 reactive	ND	130,248
Patient 28	Female	Established	Jun 2012	HIV-1 reactive	ND	78,084
Patient 58	Male	Established	Jan 2013	HIV-1 reactive	ND	67,808
Patient 61	Male	Established	Feb 2013	HIV-1 reactive	ND	65,105
Patient 29	Male	Established	Jul 2012	HIV-1 reactive	ND	49,873
Patient 24	Male	Established	Jun 2012	HIV-1 reactive	ND	44,816
Patient 48	Female	Established	Dec 2012	HIV-1 reactive	ND	27,125
Patient 41	Male	Established	Oct 2012	HIV-1 reactive	ND	20,692
Patient 38	Male	Established	Sep 2012	HIV-1 reactive	ND	14,925
Patient 30	Male	Established	Jul 2012	HIV-1 reactive	ND	9,519
Patient 22	Female	Established	May 2012	HIV-1 reactive	ND	4,334
Patient 37	Male	Established	Sep 2012	HIV-1 reactive	ND	1,537
Patient 49	Female	Established	Dec 2012	HIV-1 reactive	ND	1,225
Patient 47	Female	Established	Nov 2012	HIV-1 reactive	ND	757

**Abbreviations:** HIV = human immunodeficiency virus; IA = immunoassay; ND = not done.

## Footnotes

* Maricopa Integrated Health Systems is a public health-care system in Maricopa County, Arizona, that provides hospital- and clinic-based medical care to area residents.
